# Diagnostic and prognostic values of pyroptosis-related genes for the hepatocellular carcinoma

**DOI:** 10.1186/s12859-022-04726-7

**Published:** 2022-05-13

**Authors:** Mindan Xing, Jia Li

**Affiliations:** 1 Nankai University School of Medicine, Nankai University, Tianjin, China; 2 Tianjin Second People’s Hospital, Tianjin, China

**Keywords:** Hepatocellular carcinoma, Pyroptosis, Prognostic markers, Diagnosis markers, Risk model

## Abstract

**Background:**

Due to the high heterogeneity, the early diagnosis and prognostic prediction of hepatic cellular cancer (HCC) is challenging. In this study, we explored the diagnostic and prognostic value of pyroptosis-related genes (PRGs) in HCC. We downloaded the mRNA expression profiles of HCC and the corresponding clinical data from the TCGA and ICGC databases. Fifty-one PRGs were extracted from Genecards, MsigDB, and relevant literature. The area under the receiver operating characteristic (AUC) was used to explore the diagnostic value of the PRGs.

**Results:**

The results revealed that *BAK1, BAX, CHMP2A, CHMP4C, CHMP6, GSDMC*, and *GSDMD* had higher diagnostic values for HCC (AUCs > 0.8, *P* < 0.05). Then, univariate and multivariate analyses of 51 PRGs were performed for HCC samples, and 4 PRGs (*TP53, GPX4, GSDMC, BAK1*) associated with HCC prognosis were obtained and used to construct a pyroptosis-related risk model. HCC samples were divided into high-risk and low-risk groups based on the risk score’s cut-off. Kaplan–Meier curve and Log-rank test were used to compare the overall survival (OS) of two risk groups. The OS was lower in the high-risk group than in the low-risk group. In addition, the time-dependent receiver operating characteristics revealed that the risk model could be used to predict the prognosis of HCC more accurately. The risk score also resulted as an independent risk factor for HCC prognosis (TCGA: HR = 2.45, 95% CI 1.53–3.92; ICGC: HR = 2.19, 95% CI 1.39–3.46). Moreover, the AUC of the risk score for diagnosing HCC was relatively higher (TCGA: AUC = 0.840, *P* < 0.05; ICGC: AUC = 0.795, *P* < 0.05).

**Conclusions:**

In a word, *BAK1, BAX, CHMP2A, CHMP4C, CHMP6, GSDMC, GSDMD*, and the pyroptosis-related risk model could be used to diagnose the HCC, and the risk score also resulted as an independent risk factor for the HCC prognosis.

**Supplementary Information:**

The online version contains supplementary material available at 10.1186/s12859-022-04726-7.

## Introduction

Liver cancer is one of the most common malignancies and the second most deadly cancer worldwide [1]. Hepatocellular carcinoma (HCC) accounts for 85–90% of all primary hepatocarcinoma [1, 2]. It is also the fifth most common cancer type globally and the third largest cause of cancer-related deaths worldwide [3].

Imaging, including computed tomography, magnetic resonance imaging, ultrasonography, positron emission tomography, and angiography, is the most widely used tool for liver cancer diagnosis, while other methods include biopsy and serological analysis [2]. Yet, different imaging examinations may have different sensitivity and usually require artificial intelligence processing (e.g., radiomics) for a more accurate diagnosis. Also, these methods are dependent on the professional knowledge of examiners [2]. Nuclear magnetic resonance imaging (MRI) is an imaging examination that offers high sensitivity; however, routine monitoring may be very costly [4]. On the other hand, liver biopsy and consequently pathological examination of the malignant tissue can be very accurate and can predict prognosis. However, this method is invasive and not always well tolerated by the patients [4]. Serological detection is a relatively economical and convenient monitoring method [2]. Still, the existing serum detection indicators, such as alpha-fetoprotein (AFP), alpha-fetoprotein heterosomes, and abnormal prothrombin, have lower sensitivity and specificity [2]. Therefore, it is essential to explore more candidate markers for early diagnosis and prognosis of HCC.

Pyroptosis is a form of programmed cell death [5, 6]. Morphologically, pyroptotic cell death is characterized by necrosis and apoptosis [5]. Pyroptosis is closely associated with multiple diseases, especially malignancies [7–10]. For example, alcohol accumulation could mediate the occurrence and development of esophageal cancer through pyroptosis pathways [8]. A previous study found that, in gastric cancer cells, downregulation of gasdermin D (*GSDMD*) inhibits pyroptosis and accelerates the expression of Cdk2/cyclin A2 complexes that accelerate GC cell proliferation [9]. In ovarian cancer, growth arrest-specific transcript 5 (*GAS5*) can hinder the growth of cancer cells through proptosis mediated by caspase-1 [10].

Previous studies have reported that pyroptosis has an important role in the formation and development of HCC [11, 12]. Wei et al. [13] found that the expression of NLR family pyrin domain containing 3 (*NLRP3)* in HCC tissues decreases significantly compared with normal liver tissues. Thus, pyroptosis in HCC was reduced [13]. However, the relationship of pyroptosis-related genes (PRGs) with the diagnosis and prognosis of HCC remains unclear. In the present study, we explored the diagnostic and prognostic value of PRGs in HCC, found some candidate markers for diagnosing HCC, and constructed a risk model used to diagnose and monitor liver cancer.

## Materials and methods

### Data collection

RNA sequencing data and clinical information of HCC were extracted from the TCGA (https://portal.gdc.cancer.gov/repository) and ICGC databases (https://dcc.icgc.org/releases/current/Projects/LIRI-JP). 374 HCC samples and 50 non-tumor samples were collected from TCGA, and 231 HCC samples and 194 non-tumor samples were from ICGC. To ensure reliability, genes with reading counts equaling 0 in more than 25% of the samples were removed for further analysis. We enrolled all paired samples in the TCGA and ICGC cohorts to explore the diagnostic value of the PRGs (TCGA: 50 paired samples, ICGC: 194 paired samples) (Additional file 1: Table S1). During the processing of exploration of the prognostic value of PRGs for HCC, patients whose survival time was < 0.1 months or those with incomplete information (survival or tumor stages) were excluded from the analysis. Finally, 311 HCC samples from the TCGA cohort and 231 HCC samples from the ICGC cohort were used to study the prognostic value of PRGs (Additional file 2: Table S2). All data were obtained from the TCGA and ICGC databases, so all methods were performed following the ethical guidelines of the 1975 Declaration of Helsinki.

### Pyroptosis related genes selection

Pyroptosis-related genes (PRGs) were extracted from GeneCards (https://www.genecards.org/), and six genes with a relevance score > 7 were selected. Twenty-seven pyroptosis-related genes (Reactome pyroptosis) were downloaded from the Molecular Signature Database v7.4 (MSigDB). Thirty-three pyroptosis genes were extracted from the literature [14–17]. After removing the overlapping genes, 51 PRGs were obtained for further study (Additional file 3: Table S3).

### Identification of the DEGs between the tumor and non-tumor samples

Fifty paired samples from the TCGA cohort and 194 paired samples from the ICGC cohort were used to identify the differentially expressional genes (DEGs) of PRGs. The *DESeq2* package was used to explore the DEGs with an adjusted *P* value < 0.05. Next, we analyzed the diagnostic efficacy of the DEGs for HCC using MedCalc 19.0.4 software through the area under the receiver operating characteristic curve (AUC).

### Construction of the pyroptosis‐related risk model

Three hundred and eleven tumor samples from the TCGA cohort and 231 tumor samples from the ICGC cohort were used to analyze the prognostic value of the PRGs. Firstly, we conducted univariate Cox regression analysis by using the *survival* R package to identify PRGs associated with the prognosis of the HCC in the TCGA cohort. The PRGs with *P* < 0.20 were retained. Next, stepwise regression of multivariate Cox analysis was performed to establish a risk model closely related to HCC prognosis. Stepwise regression is defined by gradually entering independent variables into the model, if the model is statistically significant, and incorporating them into the regression model. Variables that were not statistically significant were also removed. Finally, an automatic fitting regression model is obtained. The risk score was computed as follows: Risk score = $\mathop \sum \limits_{i = 1}^{N} Xi \times Yi$, where *X* is a coefficient and *Y* is a gene expression level.

### Exploration and validation of the performance of the risk model

We used the *survminer* R package to determine the optional cut-off value of the risk score in the TCGA and ICGC cohorts. The tumor samples were divided into the high-risk and low-risk groups based on the cut-off value. Kaplan‐Meier curves and Log-rank test were performed using the *survival* and *survminer R* packages to compare the overall survival (OS) of two risk groups. The R package *timeROC* was used to establish a time‐dependent receiver operating characteristic curve (ROC) to check the efficiency of the risk score in predicting the outcomes of HCC. *ggDCA* R package was used to conduct decision curve analysis (DCA) and to explore the accuracy of the risk model. In addition, *tidyverse* R package was used to perform principal component analysis (PCA) and show differences between the high-risk and low-risk groups.

### Prognostic and diagnostic value of pyroptosis‐related risk model

Univariate and multivariate Cox analyses explored the independent prognostic value of the risk score for the HCC. *Wilcoxon* test was used to test the difference of risk score between tumor and non-tumor samples. The ROC analysis was conducted to explore the diagnostic value of the risk score for the HCC in the paired samples.

### Functional analysis of the DEGs between the high-risk and low-risk groups

To understand the different functions of the high-risk and the low-risk groups, we compared all the genes expression levels between the two risk groups, selected the DEGs, and conducted gene ontology (GO) and Kyoto Encyclopedia of Genes and Genomes (KEGG) analysis [18–20]. The *DESeq2 R* package was used to compare the expression level of all genes between two risk groups and select the DEGs in the TCGA cohort. The screening criteria were |log2FC| > 1 and *P*.adjust < 0.05. PCA was used to perform the difference between the two risk groups. The heat map was used to perform the expression levels of the DEGs. Also, the volcano was used to show the numbers of the DEGs. Next, the *clusterProfiler* package was used to conduct GO and KEGG analysis [18–20]. If the *P* value was < 0.05, the enrichment was considered statistically significant.

### Comparison of the immune status between two risk groups

The *ESTIMATE* R package was used to calculate the immune scores, the stromal scores, and the tumor purity. The *gsva* R package was used to conduct the single sample gene set enrichment analysis (ssGSEA), calculate the scores of the immune cell subtypes, and evaluate the activity of immune-related pathways. Tumor stem cell features extracted from transcriptome and epigenetics of TCGA tumor samples were used to measure stem cell-like features of the tumor. The immune cell subtypes related gene set was shown in Additional file 4: Table S4, and the gene set associated with the immune-related pathway was shown in Additional file 5: Table S5 [21, 22]. *Wilcoxon* test was used to compare the tumor stemness between the two risk groups. *Spearman* correlation analysis was used to analyze the association of tumor stemness with the risk score.

### Acquisition of immunotherapeutic cohorts

To assess the predictive value of risk score for the efficacy of immunotherapy, we cited the IMvigor210 cohort (Additional file 6: Table S6) [23], which investigated the efficacy of anti-PD-L1 antibody (pembrolizumab) in patients with advanced urothelial cancer (http://research-pub.Gene.com/imvigor210corebiologies). The complete transcriptome data and clinical information were enrolled in the present study.

### Statistical analysis

*One-way ANOVA* and *Kruskal–Wallis* tests [24] were used for multiple comparisons. *Wilcoxon* test was used to test the differences between the two groups. Kaplan–Meier curve was used to generate survival curves, and the significance of differences was compared using the Log-rank test. Hazard ratios (HRs) and 95% Confidence Interval (CI) were calculated using univariate and multivariate Cox analyses. All statistical *P* values were two-sided. A *P* < *0.05* was considered to be statistically significant. The R 4.1.1, SPSS 23.0, and MedCalc.19.7.2 software was used to perform all data processing.

## Results

### Identification of pyroptosis-related DEGs between tumor and non-tumor samples

**Fig. 1:**
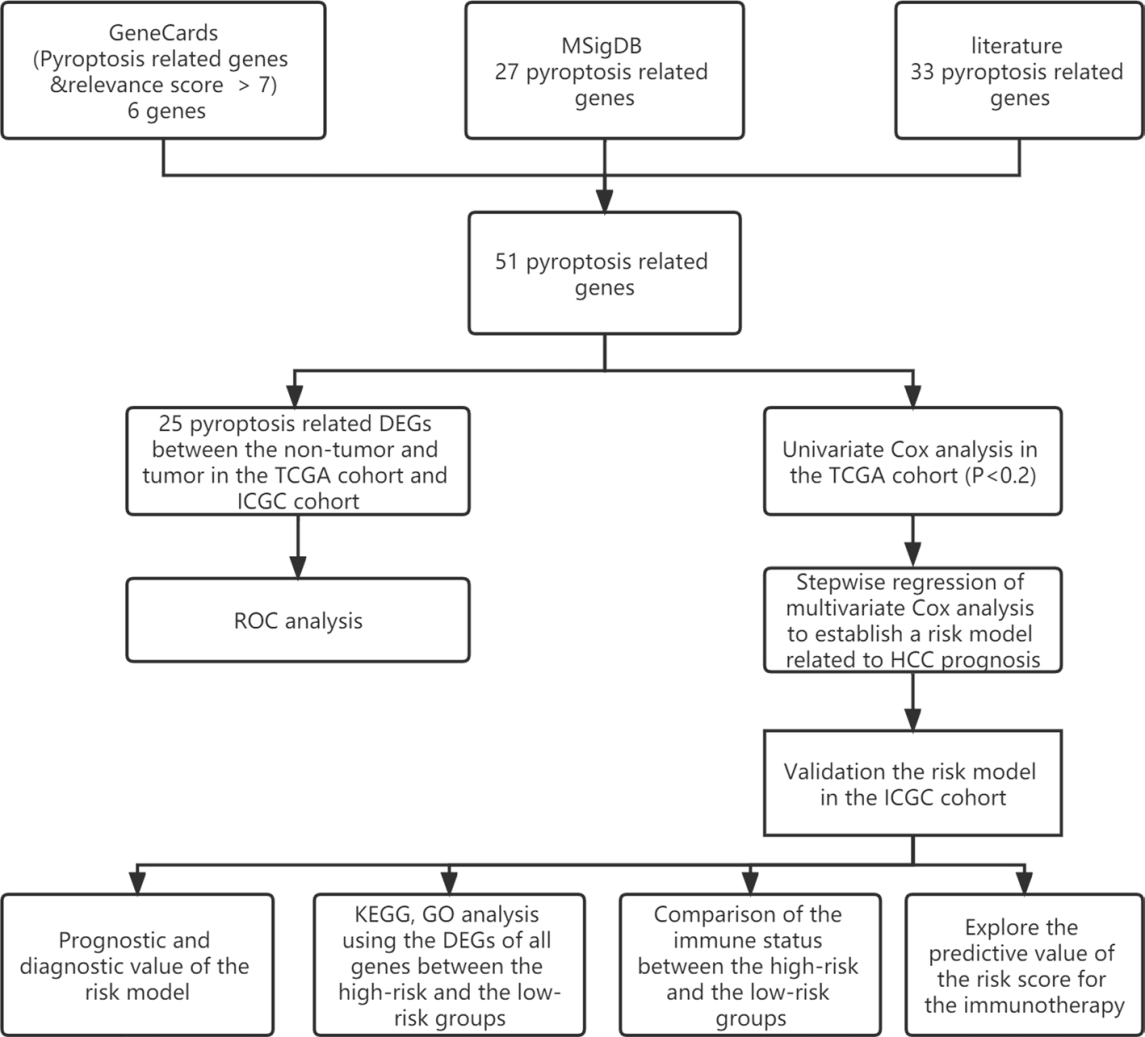
The workflow chart in this study

**Fig. 2:**
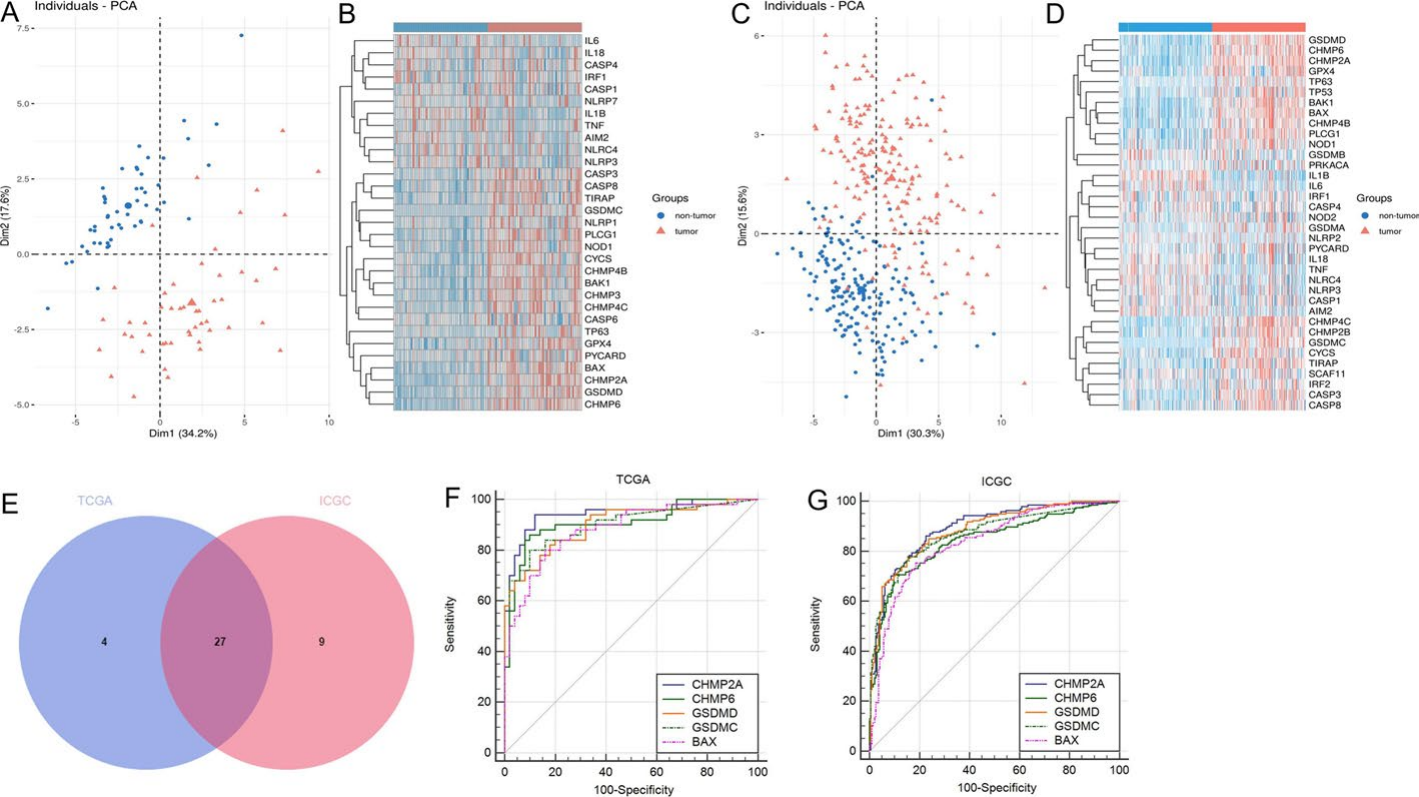
Identification of pyroptosis-related DEGs between tumor and non-tumor samples. **A**, **C** Principal component analysis plot (PCA) based on the DEGs (**A**: TCGA cohort; **D**: ICGC cohort). **B**, **D** Heatmap displaying different expressions of the DEGs. Red indicates higher expression, and blue represents a lower expression (**B**: TCGA cohort; **D**: ICGC cohort). **E** A Venn diagram shows the number of overlapped DEGs in two cohorts. **F**, **G** The receiver operator characteristic curve (ROC) of *CHMP2A, CHMP6, GSDMD, GSDMC, BAX* in paired samples of the TCGA and ICGC cohorts (**H**: TCGA cohort; **I**: ICGC cohort)

The workflow chart is shown in Fig. 1. In the TCGA cohort, 31 out of 51 PRGs were significantly different in tumor and non-tumor samples (Fig. 2A, Additional file 7: Table S7); their expression levels are shown in Fig. 2B. In the ICGC cohort, 36 out of 51 PRGs were significantly different in tumors and non-tumor samples (Fig. 2C, D, Additional file 8: Table S8). There were 27 overlapping DEGs between the TCGA cohort and the ICGC cohort (Fig. 2E); 10 DEGs (*IL1B, NLRC4, IL6, NLRP3, IL18, TNF, IRF1, AIM2, CASP4, CASP1*) were downregulated and 17 DEGs (*GSDMC, BAK1, PLCG1, BAX, GSDMD, CASP8, TP63, CYCS, PYCARD, NOD1, CHMP4C, CHMP6, CHMP2A, TIRAP, CHMP4B, CASP3, GPX4*) were upregulated in tumor samples.

### The diagnostic value of the DEGs for HCC

Next, we analyzed the diagnostic efficacy of the 27 pyroptosis-related DEGs for HCC by ROC analysis (Table 1). The AUCs of 7 pyroptosis-related DEGs were > 0.8 (*P* < 0.05) in the TCGA and ICGC cohorts, including *BAK1, BAX, CHMP2A, CHMP4C, CHMP6, GSDMC,* and *GSDMD*. The ROC of the DEGs with the AUC that ranked in the top 5 are shown in Fig. 2F, G.

**Table 1:** The AUC of the DEGs between the TCGA cohort and ICGC cohort

Symbol	TCGA		ICGC	
	AUC	*P*	AUC	*P*
AIM2	0.665	0.005	0.619	< 0.001
BAK1	0.864	< 0.001	0.831	< 0.001
BAX	0.882	< 0.001	0.835	< 0.001
CASP1	0.508	0.885	0.537	0.213
CASP3	0.801	< 0.001	0.779	< 0.001
CASP4	0.563	0.279	0.529	0.325
CASP8	0.828	< 0.001	0.696	< 0.001
CHMP2A	0.945	< 0.001	0.892	< 0.001
CHMP4B	0.852	< 0.001	0.79	< 0.001
CHMP4C	0.815	< 0.001	0.817	< 0.001
CHMP6	0.908	< 0.001	0.841	< 0.001
CYCS	0.86	< 0.001	0.72	< 0.001
GPX4	0.820	< 0.001	0.718	< 0.001
GSDMC	0.899	< 0.001	0.868	< 0.001
GSDMD	0.901	< 0.001	0.883	< 0.001
IL18	0.613	0.051	0.601	0.001
IL1B	0.802	< 0.001	0.78	< 0.001
IL6	0.651	0.009	0.678	< 0.001
IRF1	0.534	0.563	0.52	0.506
NLRC4	0.602	0.08	0.603	< 0.001
NLRP3	0.625	0.031	0.601	0.001
NOD1	0.805	< 0.001	0.666	< 0.001
PLCG1	0.85	< 0.001	0.787	< 0.001
PYCARD	0.617	0.043	0.581	0.006
TIRAP	0.806	< 0.001	0.770	< 0.001
TNF	0.640	0.016	0.668	< 0.001
TP63	0.684	0.002	0.646	< 0.001

### Construction and validation of the pyroptosis-related risk model

**Fig. 3:**
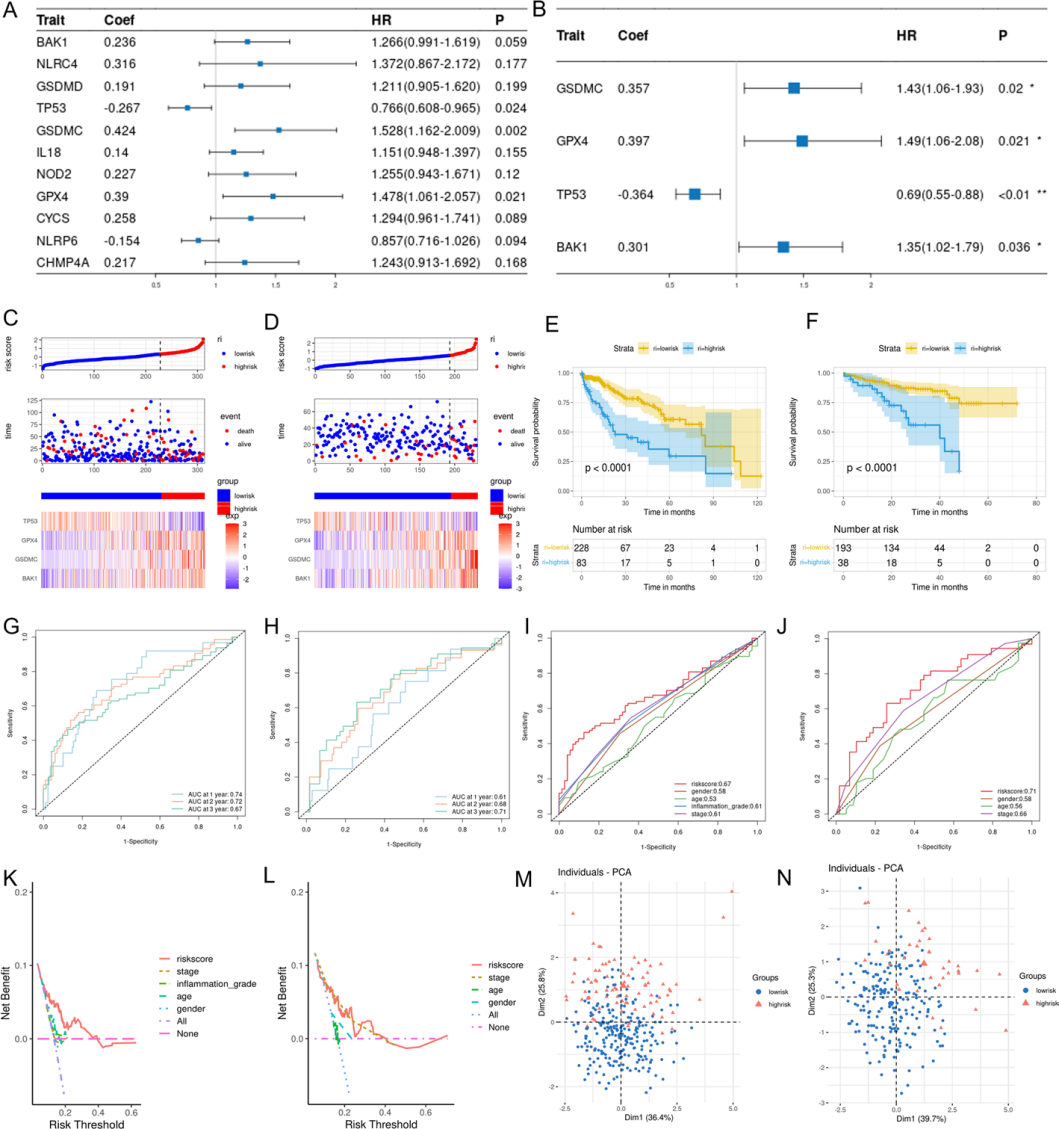
Construction and validation of the pyroptosis-related risk model. **A** Eleven PRGs screened by univariate Cox analysis were associated with HCC prognosis in the TCGA cohort (*P* < 0.20). **B** The multivariate Cox analysis of the 4 PRGs used to construct the risk score. **C**, **D** The proportion of deaths and the expression of the 4 PRGs changed in high-risk and low-risk groups as risk scores increased. Red, upregulated PRGs; blue, downregulated PRGs. (**C**: TCGA cohort; **D**: ICGC cohort). **E**, **F** The Kaplan–Meier curves of the OS in the high-risk and low-risk groups and *P* value obtained by log-rank test. Blue represents the high-risk group, and yellow, the low-risk group. (**E**: TCGA cohort; **F**: ICGC cohort). **G**, **H** The time-dependent ROC was used to display the predictive efficiency of the risk score for HCC prognosis at different times. (**G**: TCGA cohort, **H**: ICGC cohort). **I**, **J** The ROC curve was used to display the predictive efficiency of risk score and other clinical information for patient 3-year survival. (**I**: TCGA cohort, **J**: ICGC cohort). **K**, **L** The DCA was used to analyze the accuracy of the risk score for HCC prognosis. (**K**: TCGA cohort, **L**: ICGC cohort) **M**, **N** PCA for the 4 PRGs revealed the high-risk group and the low-risk group could be distributed in different regions. Blue, low-risk group; Red, high-risk group. (**M**: TCGA cohort, **N**: ICGC cohort)

Using univariate Cox analysis, we found the expressions of *BAK1, CYCS, CHMP4A, NLRP6, NLRC4, NOD2, GPX4, GSDMD, GSDMC, TP53,* and *IL18* were related to the prognosis of HCC with P < 0.20 in the TCGA cohort (Fig. 3A). Next, we constructed a pyroptosis-related risk model associated with HCC prognosis using the stepwise regression method in the multivariate COX analysis. The risk model was created based on the expressions of *TP53, GPX4, GSDMC,* and *BAK1* (Fig. 3B). The risk score was estimated as follows: Risk score = (0.357*expression level of GSDMC) + (0.397*expression level of *GPX4*) + (0.301* expression level of *BAK1*) + (− 0.364*expression level of *TP53*). Among them, *GSDMC, GPX4, and BAK1* were associated with increased risk (*GSDMC*: HR = 1.430, 95% CI 1.06–1.93; *GPX4*: HR = 1.490, 95% CI 1.060–2.080; *BAK1*: HR = 1.350, 95% CI 1.020–1.790), and *TP53* was a protective gene (*TP53*: HR = 0.690, 95% CI (0.550–0.880). The C‐index for the TCGA and the ICGC cohorts was 0.682 and 0.657, respectively.

In the TCGA cohort, the risk score's cut-off value (0.344) was determined by *survminer* R package. Consequently, 311 tumor samples were divided into the high-risk and low-risk groups based on the cut-off value. In the ICGC cohort, we calculated the risk score according to the same risk model constituted by four genes. And the cut-off value (0.571) of the risk score was determined by *survminer* R package. 231 tumor samples were divided into the high-risk and low-risk groups based on the cut-off value. The proportion of dead patients was higher, and the survival time was shorter in the high-risk group of the TCGA and ICGC cohorts (Fig. 3C, D). In addition, as the risk score increased, the expression of *GSDMC, GPX4,* and *BAK1* gradually increased, and the expression of *TP53* gradually decreased (Fig. 3C, D).

The result of the Log-rank test revealed the OS was lower in the high-risk group compared to the low-risk group (*P* < 0.05, Fig. 3E, F). In the TCGA cohort, the AUC of the risk score was 0.74 for 1-year, 0.72 for 2-year, and 0.67 for 3-year survival (Fig. 3G). In the ICGC cohort, the AUC was 0.61 for 1-year, 0.68 for 2-year, and 0.73 for 3-year survival (Fig. 3H). In addition, the AUC of the risk score was larger than other clinical features in the two cohorts (Fig. 3I, J). Also, the result of the DCA suggested that the risk score was more accurate in evaluating the prognosis of HCC than other clinical features in the two cohorts (Fig. 3K, L). In addition, the results of the PCA in the two cohorts suggested that the high-risk group and the low-risk group were distributed in different regions (Fig. 3M, N).

### Prognostic value and diagnostic value of pyroptosis‐related risk model

**Fig. 4:**
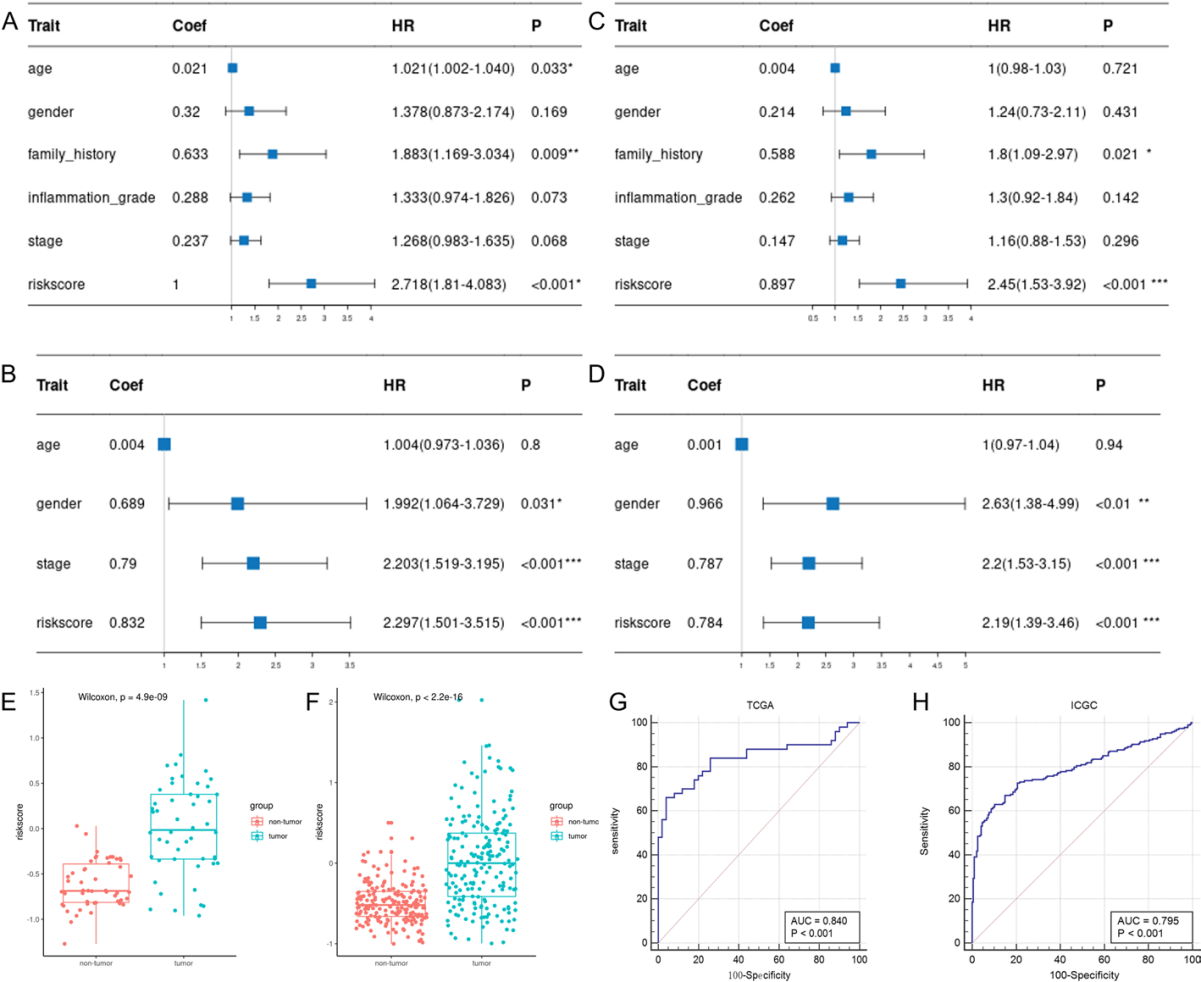
Prognostic and Diagnostic value of pyroptosis-related risk model. **A**, **B** Univariate Cox analysis for the risk score and other clinical features. (**A** TCGA cohort, **B** ICGC cohort). **C**, **D** Multivariate Cox analysis for the risk score and other clinical features. (**C** TCGA cohort; **D** ICGC cohort). **E**, **F** Comparison of the risk scores in paired samples of the two cohorts. Red, non-tumor samples; green, tumor samples. (**E** TCGA cohort; **F** ICGC cohort). **G**, **H** The ROC of a risk score for the diagnosis of HCC in two cohorts (**G** TCGA cohort; **H** ICGC cohort)

In the TCGA and the ICGC cohorts, the results of the univariate Cox analysis revealed that the risk scores were significantly correlated with OS (TCGA cohort: HR = 2.718, 95% CI = 1.810–4.083, *P* < 0.001; ICGC cohort: HR = 2.297, 95% CI = 1.501–3.515, *P* < 0.001) (Fig. 4A, B). After adjusting for confounding factors, multivariate Cox analysis showed that the risk score was an independent risk factor for the OS (TCGA cohort: HR = 2.450, 95% CI = 1.530–3.920, *P* < 0.001; ICGC cohort: HR = 2.190, 95% CI = 1.390–3.460, *P* =  < 0.01) (Fig. 4C, D).

In the paired samples of the TCGA and the ICGC cohorts, the risk scores were significantly higher in the tumor samples than in the non-tumor samples (*P* < 0.05, Fig. 4E, F). In the TCGA cohort, the AUC of the risk score for diagnosing HCC was 0.840 (*P* < 0.001, Fig. 4G). In the ICGC cohort, the AUC of the risk score for diagnosing HCC was 0.795 (*P* < 0.001, Fig. 4H).

### Correlation of the pyroptosis‐related risk score with clinicopathologic features

**Fig. 5:**
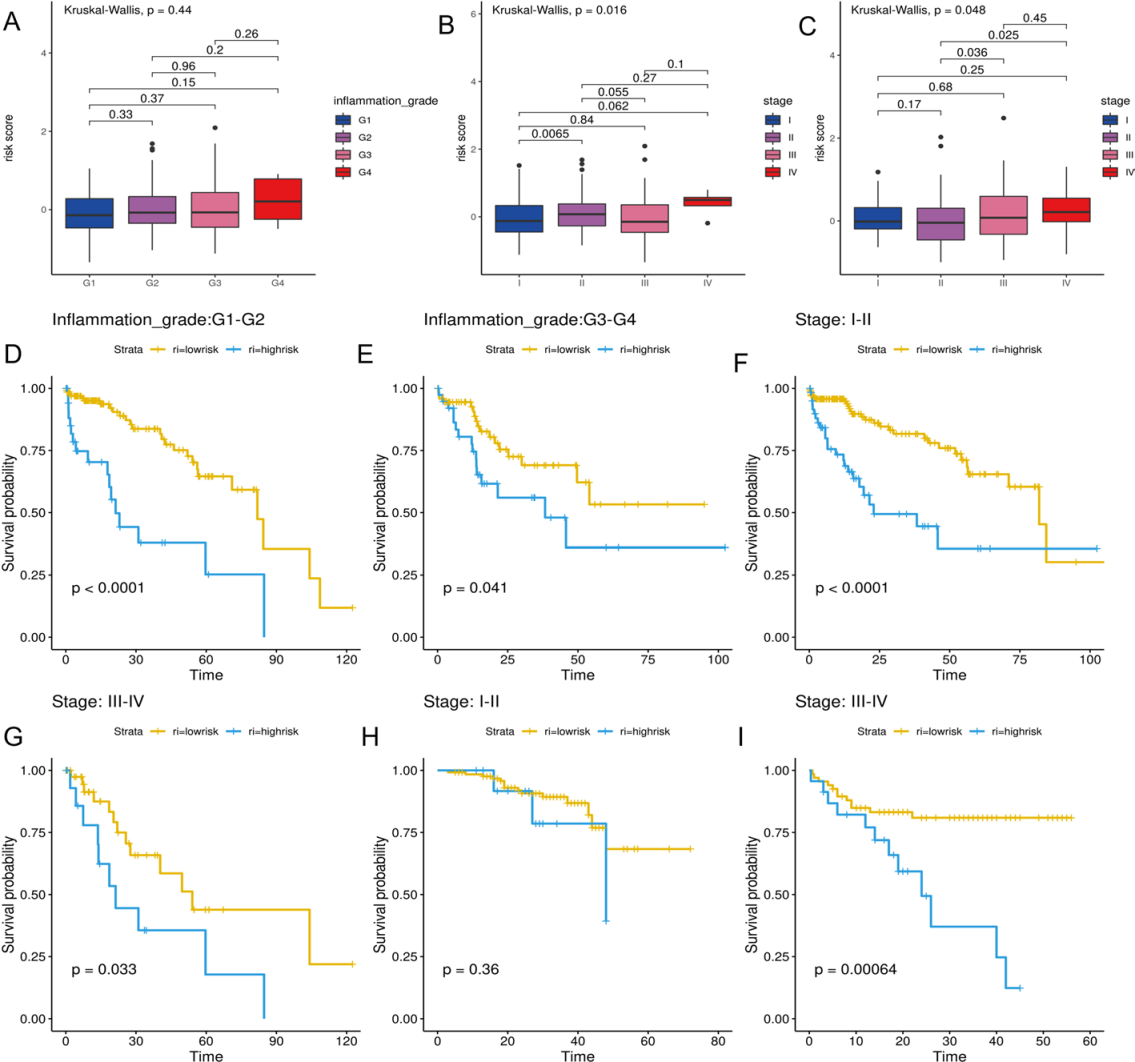
Correlation of the pyroptosis‐related risk score with clinicopathologic features. **A** The difference among different inflammation grades in the TCGA cohort. **B** The difference of the risk score among different tumor stages in the TCGA cohort. **C** The difference of the risk score among different tumor stages in the ICGC cohort. **D**–**G** Kaplan–Meier curves for the OS of patients with different clinicopathologic in the TCGA cohort. **H**, **I** Kaplan–Meier curves for the OS of patients with different clinicopathologic in the ICGC cohort

The risk scores increased with inflammation grade, although these differences were not statistically significant in the TCGA cohort (*P* > 0.05, Fig. 5A). However, in both cohorts, the differences in the risk scores among tumor stages were statistically significant, and risk scores gradually increased with tumor stages (*P* < 0.05, Fig. 5B, C).

Next, we compared the OS between the high-risk and low-risk groups in HCC with different clinical features using the Log-rank test. In patients with different clinicopathologic features of the TCGA cohorts, the OS was significantly lower in the high-risk group than in the low-risk group (*P* < 0.05, Fig. 5D–G). In the patients with stage I/II of ICGC cohort, the difference in the OS between the two risk groups was not statistically significant (*P* > 0.05), which may be related to the small death toll (Fig. 5H). Yet, in patients with stage III/IV from ICGC cohort, the OS was lower in the high-risk group than in the low-risk group (*P* < 0.05, Fig. 5I).

### Functional enrichment analysis of the DEGs between the high-risk and low-risk groups

**Fig. 6:**
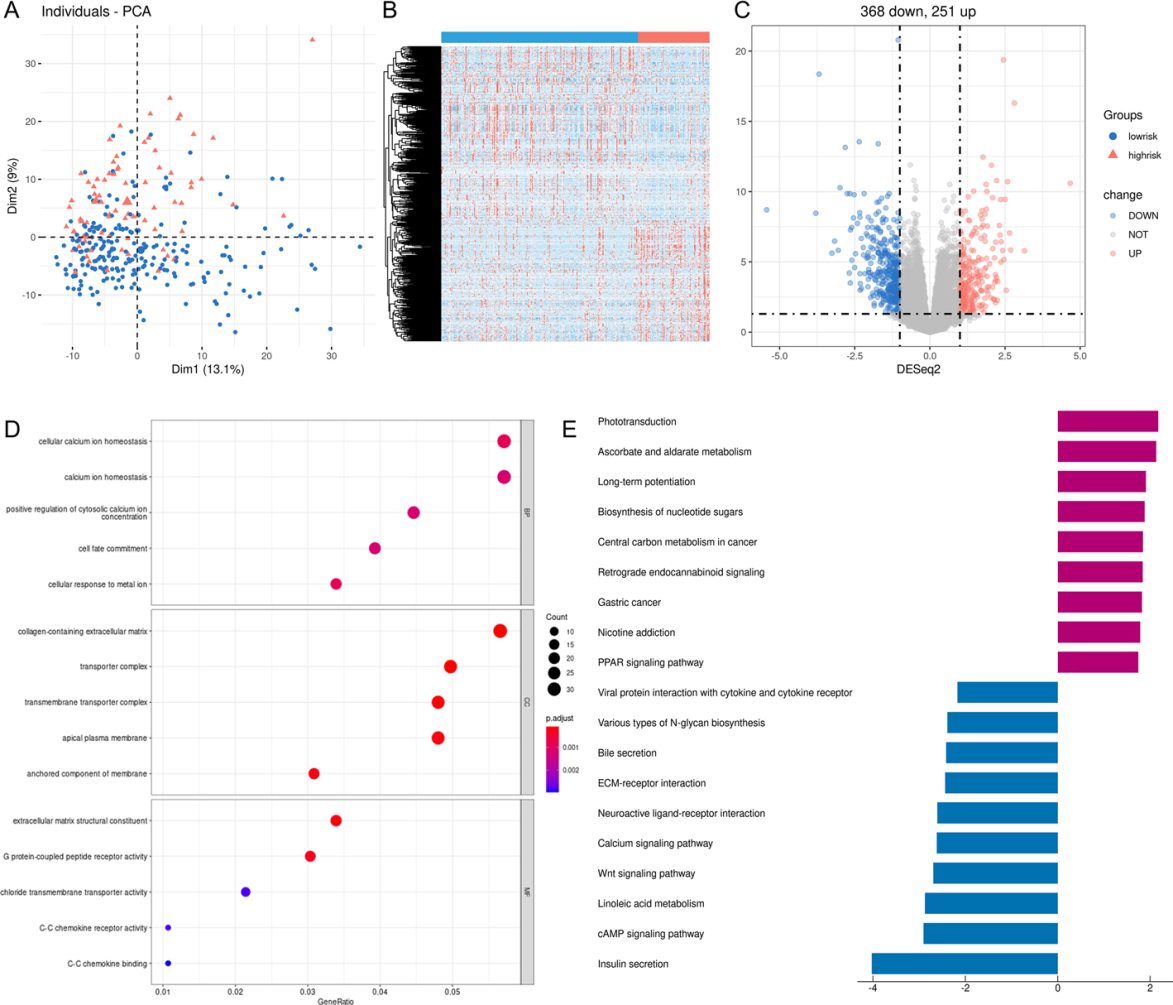
Identification and functional enrichment analysis of the DEGs between the two risk groups in the TCGA cohort. **A** PCA plot based on the DEGs. **B** The heatmap shows the expression level of the DEGs between the two risk groups. Red indicates higher expression, and blue, lower expression. **C** The volcano map shows the number of upregulated and downregulated genes in the high-risk group. Blue represents downregulated genes, and red denotes upregulated genes. **D** The bubble graph for GO enrichment analysis shows the function of the DEGs. BP: biological process; CC, cell component; MF, molecular function. The bigger bubble indicates the more genes enriched, and the increasing depth of red indicates the differences were more obvious. **E** The barplot graph for KEGG pathways. The left bar indicates the pathways enriched by the downregulated DEGs, and the right bar indicates the pathways enriched by the upregulated DEGs. The longer bar indicates that the differences were more prominent

In the TCGA cohort, we compared the expression level of all genes between the two risk groups and identified 619 DEGs using *DESeq2* R packages according to specific criteria (|log2FC| > 1 and *P*.adjust < 0.05). PCA based on the DEGs showed that the two risk groups were distributed in different regions and revealed significant differences between the two risk groups (Fig. 6A). The heatmap suggested that the expression levels of the DEGs were obviously different (Fig. 6B). Among 619 DEGs, 368 were downregulated, and 251 were upregulated in the high-risk group (Fig. 6C).

Functional annotations of GO enrichment indicated these DEGs were significantly associated with the regulation of calcium ions, such as “cellular calcium ion homeostasis”, “calcium ion homeostasis”, and “positive regulation of cytosolic calcium ion concentration” (Fig. 6D). KEGG pathway analysis [18–20] demonstrated that these DEGs were correlated with the formation of cancers and calcium, such as “Central carbon metabolism in cancer”, “Gastric cancer”, and “Calcium signaling pathway” (Fig. 6E).

### Immune status and tumor microenvironment analysis

**Fig. 7:**
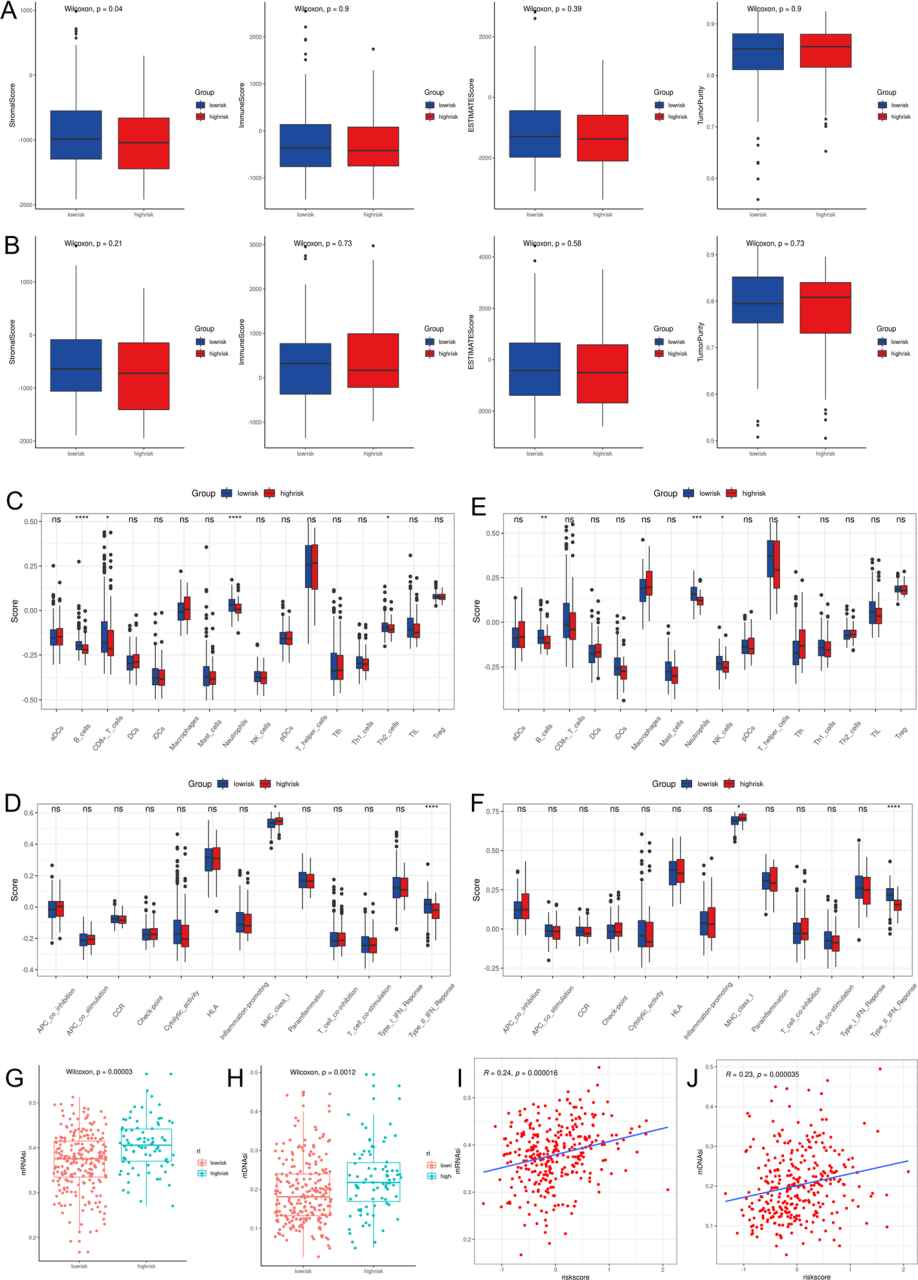
Comparison of immune status between the high-risk and low-risk groups. **A**, **B** Comparison of the stromal scores, immune scores, ESTIMATE scores, and tumor purity. (**A** TCGA cohort; **B** ICGC cohort). **C** The differences of the immune cell subtypes between the two risk groups in the TCGA cohort. **D** The differences of the immune-related pathways between the two risk groups in the TCGA cohort. **E** The differences of the immune cells subtypes between the two risk groups in the ICGC cohort. **F** The differences of the immune-related pathways between the two risk groups in the ICGC cohort. **G**, **H** Comparison of mRNAsi and mDNAsi of the high-risk and the low-risk groups in the TCGA cohort. (**G** mRNAsi; **H** mDNAsi). **I**, **J** The relationship of mRNAsi and m DNAsi with the risk score in the TCGA cohort. (**I** mRNAsi; **J** mDNAsi) (*P < 0.05, **P < 0.01, *** *P* < 0.001, ns *P* > 0.05)

To further explore the correlation between risk score and immune status, we used the *Estimate* package to calculate the purity of the tumor and the immune scores of the tumor microenvironment. The results revealed no statistically significant differences (*P* > 0.05) in tumor purity and immune score between the high-risk and low-risk groups of TCGA and ICGC cohorts (Fig. 7A, B).

The enrichment scores of different immune cell subtypes and immune-related pathways were quantified by ssGSEA. We found that B cells, CD8 + T cells, neutrophils, and Th2 cells were reduced (*P* < 0.05, Fig. 7C), and the “MHC class I” and “Type II IFN Response” were downregulated in the high-risk group of the TCGA cohorts (Fig. 7D). Similar results were obtained in the ICGC cohort (Fig. 7E, F).

Tumor stemness can be measured by RNA stemness score (mRNAsi) based on mRNA expression and DNA stemness score based on DNA methylation pattern (mDNAsi) [24]. mRNAsi and mDNAsi were significantly higher in the high-risk group than in the low-risk group of the TCGA cohort (*P* < 0.05, Fig. 7G, H). In addition, the mRNAsi and mDNAsi were positively correlated with the risk score (mRNAsi: r = 0.24, *P* < 0.05; mDNAsi: r = 0.23, *P* < 0.05, Fig. 7I, J).

### Assessment of the predictive value of the risk score for immunotherapy efficacy

**Fig. 8:**
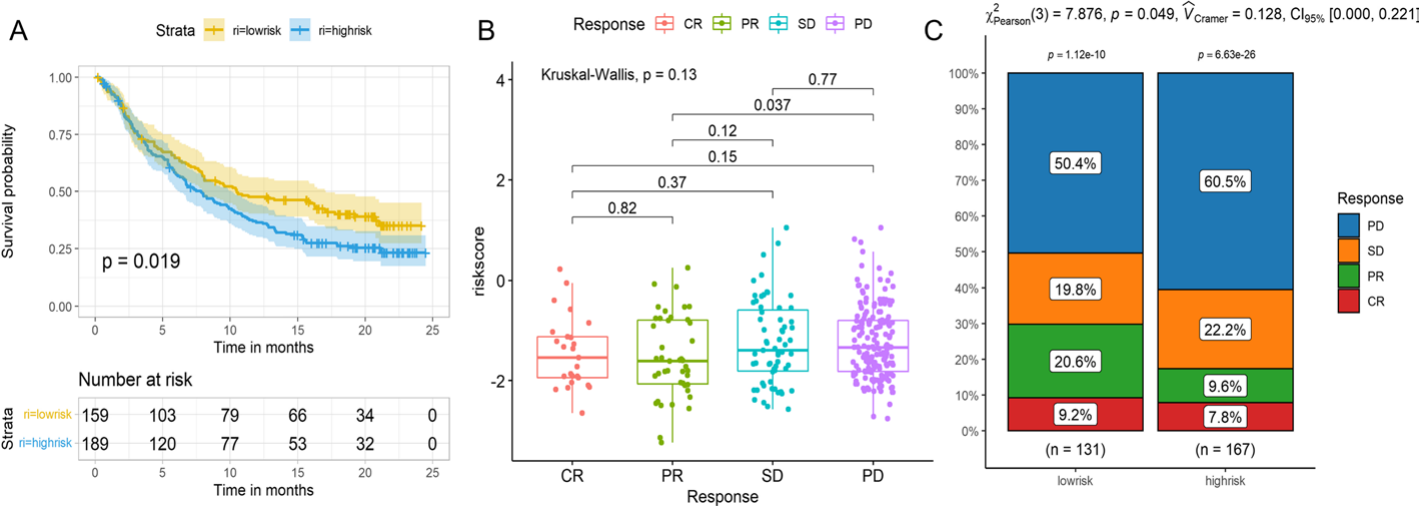
Assessment of the predictive value of risk score for immunotherapeutic responses. **A** Kaplan–Meier curves for the OS of patients in the IMvirgor210 cohort. **B** The difference of the risk score among different anti-PD-L1 clinical response groups. **C** The proportion of patients responding to PD-L1 blockade therapy in the high-risk and low-risk groups. (CR: complete response, PR: partial response, SD: stable disease, PD: a progressive disease)

Finally, we used the IMvirgor210 cohort to explore whether the risk score could assess the effect of immunotherapy. And the risk score was calculated according to the risk model constitute by four PRGs. The *survminer* R package determined the cut-off value (− 1.539). The low-risk group showed a significant clinical benefit and obviously longer survival (*P* < 0.05, Fig. 8A). The difference of the risk score among the patients with complete response (CR), partial response (PR), stable disease (SD), and progressive disease (PD) was not statistically significant (*P* > 0.05, Fig. 8B). However, the risk score was lower in patients with CR and PR than in those with SD and PD (*P* > 0.05, Fig. 8B). The risk score was the highest in the patients with PD (Fig. 8B). Also, the percent of the patients with PD was higher in the high-risk group than in the low-risk group (*P* < 0.05; Fig. 8C).

## Discussion

Recent studies have reported that cell pyroptosis has a vital role in liver disease progression [12, 13, 25]. The liver is tightly linked to the intestine through the venous system of the portal circulation. When the gut-liver axis is altered, bacteria enter the portal circulation and induce pyroptosis in hepatocytes through the bacterial lipopolysaccharides (LPS) [25]. In addition, studies have shown that liver fibrosis, cirrhosis, and HCC are strongly associated with pyroptosis [3, 26–28]. Although previous studies reported the pyroptosis in HCC is reduced [12, 13], the diagnostic and prognostic values of PRGs in HCC remain unclear. Thus, in this study, we explored whether PRGs could be used as candidate markers for diagnosing and monitoring liver cancer.

Next, we explored the diagnostic value of the 27 pyroptosis-related DEGs in paired samples of the TCGA and ICGC cohorts. The results suggested that *BAK1, BAX, CHMP2A, CHMP4C, CHMP6, GSDMC, and GSDMD* have high diagnostic values in predicting HCC (AUC > 0.8, *P* < 0.05 in both cohorts). Among them, *BAK1* and *BAX* are the critical molecules in the regulation of apoptosis [29]. Under certain conditions, apoptosis can be converted to pyroptosis [30], so *BAK1* and *BAX* can influence pyroptosis by modulating apoptosis [31]. *GSDMC, GSDMD, CHMP2A, CHMP4C,* and *CHMP6* have an essential role in both pyroptosis and apoptosis [29]. *GSDMC* and *GSDMD*, as members of *GSDM* family proteins, regulate the switch between pyroptosis and apoptosis [30]. *CHMP2A, CHMP4C,* and *CHMP6* are part of the endosomal sorting complex required for transport (ESCRT) [32]. During pyroptosis, cell membrane damage and repair are mediated by the ESCRT [32]. In this study, the above PRGs were significantly different between tumor and non-tumor samples, which suggested that pyroptosis is associated with HCC. Thus these PRGs could be used as candidate markers for the diagnosis of HCC. We could use these RNAs and proteins expressed by these genes to diagnose the HCC in the tumor tissues in a clinical setting. Importantly, these RNAs and proteins expressed by these genes in the plasma may become non-invasive candidate markers for diagnosing HCC.

To further explore the relationship of PRGs with HCC prognosis, we analyzed 51 PRGs using univariate and multivariate Cox analyses. A pyroptosis-related risk model was constructed based on *GSDMC, BAK1, TP53*, and *GPX4*. Among them, *BAK1, GSDMC* belonged to the pyroptosis-related DEGs. *TP53* can regulate pyroptosis by inducing glycolysis and apoptosis regulator in brain injury [33]. Still, further validation is needed on whether *TP53* could affect pyroptosis through apoptosis in HCC. Moreover, *GPX4*, an antioxidant defense enzyme active in repairing oxidative damage to lipids, is an important negative regulator of macrophage pyroptosis [34]. Previous studies reported that *GPX4* is involved in the development of HCC by affecting ferroptosis [35, 36]. However, whether pyroptosis affects HCC through GPX4 requires further validation.

The risk score obtained according to the pyroptosis-related risk model was strongly associated with the OS of the patient with HCC. The higher the risk score, the worse the patient's prognosis. The time-dependent ROC and DCA analysis results revealed the risk score with relatively good accuracy for predicting OS. Univariate and multivariate Cox analysis suggested that the risk score was an independent risk factor for HCC prognosis. Besides, the risk score had a high diagnostic value for HCC. Therefore, we could calculate the risk score using the RNA expression levels of the four genes in the tissues to diagnose HCC and evaluate the prognosis of HCC.

Next, the functional analysis of the DEGs between different risk groups revealed that the DEGs were mainly involved in the formation and development of cancer, the regulation of calcium ions, and the change of membrane. The regulation of calcium ions and membrane change are essential processes in pyroptosis [30].

We also observed the immune status and tumor microenvironment of the high-risk and low-risk groups. The results showed that the differences in immune scores and tumor purity between the two risk groups were not statistically significant. However, in the high risk group, some immune cells were reduced, such as neutrophils, B cells, CD8 + T cells, and Th2_cells, and some immune-related pathways were downregulated, such as “Type II IFN Response” and “MHC class I”. These findings revealed lower immune levels in the high-risk group, which may be one of the reasons for the poor prognosis in the high-risk patients [37].

Tumor stemness, including mRNAsi and mDNAsi, was used to evaluate similarity between tumor cells and stem cells [38]. mRNAsi was used to assess the similarity of gene expression between tumor cells and stem cells. mDNAsi was used to examine the epigenetic similarity between tumor cells and stem cells. These indices ranged from 0 to 1; when the index was closer to 0, it indicated a lower similarity between tumor cells and stem cells, and when it was closer to 1, the similarity was higher. In this study, mRNAsi and mDNAsi increased with bigger risk scores, which indicated that the poor prognosis in the high-risk group may be related to the high similarity between tumor cells and stem cells or may be associated with the high dedifferentiation of the tumor cells.

Finally, due to the relationship of the risk score with immune status in this study, we sought to explore its predictive value for the efficacy of immunotherapy using the IMvigor210 cohort [19]. The results revealed that a higher risk score was associated with the worse efficacy of the anti-PD-L1 antibody immunotherapy. The principle of anti-PD-L1 antibody immunotherapy is to block the combination of PD-L1 and PD-1, changing the inherent connection of immune cells and tumor cells, which eventually changes the tumor microenvironment, and stimulates the huge potential of immune cells to attack the tumor [39]. In the high-risk group, the immune cells were reduced and insufficient to kill tumor cells, resulting in a higher risk score and the worse efficacy of immunotherapy. This also suggested that the risk score could partly predict the immunotherapy efficacy of anti-PD-L1 antibody.

To the best of our knowledge, this is the first study that reported the diagnostic and prognostic values of PRGs in HCC patients. The pyroptosis-related risk model was not only an independent risk factor for HCC patients but could also be used for HCC diagnosis. We could also predict the efficacy of anti-PD-L1 antibody immunotherapy by the risk score. However, the present study has several limitations. First, this risk model must be confirmed using prospective experimental data. Second, we failed to validate the predictive value of the risk model on the immunotherapy efficacy within an HCC-related immunotherapy cohort due to the fewer data on HCC related immunotherapy cohort.

## Conclusions

Our data suggest that some PRGs have high diagnostic value for HCC. The pyroptosis-related risk model developed in this study can be used to diagnose HCC, predict the prognosis of HCC, evaluate immune cell infiltration status in the tumor microenvironment and assess the efficacy of immunotherapy to guide immunotherapy. In our future studies, we will further detect the above PRGs in plasma to analyze the diagnostic value of PRGs in plasma for HCC and establish a prospective cohort study to verify this risk model's diagnostic efficacy and prognostic evaluation value for HCC.

## Supplementary Information

**Additional file 1.** **Table S1.** The clinical information of the paired samples in the TCGA cohort and ICGC cohort.

**Additional file 2.** **Table S2.** The clinical information of HCC samples in the TCGA cohort and the ICGC cohort.

**Additional file 3.** **Table S3.** Pyroptosis-related genes.

**Additional file 4.** The gene set associated with the immune cell subtypes.

**Additional file 5.** The gene set associated with the immune-related pathways.

**Additional file 6.** **Table S6.** The clinical information of the IMvirgor210 cohort.

**Additional file 7.** The pyroptosis related DEGs between the non-tumor and tumor samples in the TCGA cohort.

**Additional file 8.** The pyroptosis related DEGs between the non-tumor and tumor samples in the ICGC cohort.

## Data Availability

Six PRGs were selected from GeneCards (https://www.genecards.org/), and 27 PRGs were downloaded from MsigDB (https://www.gsea-msigdb.org/gsea/msigdb/). The datasets generated and analyzed during the current study are available in the https://portal.gdc.cancer.gov/ and https://dcc.icgc.org/projects/LIRI-JP. The code used in this study is also available from the https://github.com/jmzeng1314/for_paper/tree/main/pyroptosis_R_code.
