# Supplementary material for: Diagnostic and prognostic values of pyroptosis-related genes for the hepatocellular carcinoma
Source: BMC Bioinformatics. 2022 May 13;23:177. doi: 10.1186/s12859-022-04726-7 (PMC9101834; doi:10.1186/s12859-022-04726-7)
Supplement: Supplementary file 1 — Additional file 1. Table S1. The clinical information of the paired samples in the TCGA cohort and ICGC cohort. [file 12859_2022_4726_MOESM1_ESM.docx]

Table S1 The clinical information of the paired samples in the TCGA cohort and ICGC cohort.

| Characteristics | TCGA | ICGC | Total |
| --- | --- | --- | --- |
|  | (N=50) | (N=194) | (N=244) |
| Gender |  |  |  |
| Male | 28 (56.0%) | 140 (72.2%) | 168 (68.9%) |
| Female | 22 (44.0%) | 54 (27.8%) | 76 (31.1%) |
| Age |  |  |  |
| Mean (SD) | 62.1 (16.1) | 67.2 (10.2) | 66.2 (11.7) |
| Median [Min, Max] | 68.5 [21.0, 79.0] | 68.5 [31.0, 86.0] | 68.5 [21.0, 86.0] |
| Missing | 2 (4.0%) | 0 (0%) | 2 (0.8%) |
| Age_group |  |  |  |
| Younger < 65 | 20 (40.0%) | 67 (34.5%) | 87 (35.7%) |
| Older > 65 | 28 (56.0%) | 127 (65.5%) | 155 (63.5%) |
| Missing | 2 (4.0%) | 0 (0%) | 2 (0.8%) |
| Family_history |  |  |  |
| NO | 21 (42.0%) | 0 (0%) | 21 (8.6%) |
| YES | 24 (48.0%) | 0 (0%) | 24 (9.8%) |
| Missing | 5 (10.0%) | 194 (100%) | 199 (81.6%) |
| Inflammation_grade |  |  |  |
| G1 | 5 (10.0%) | 0 (0%) | 5 (2.0%) |
| G2 | 27 (54.0%) | 0 (0%) | 27 (11.1%) |
| G3 | 15 (30.0%) | 0 (0%) | 15 (6.1%) |
| G4 | 0 (0.0%) | 0 (0%) | 0 (0%) |
| Missing | 3 (6.0%) | 194 (100%) | 197 (80.7%) |
| Stage |  |  |  |
| I | 18 (36.0%) | 25 (12.9%) | 43 (17.6%) |
| II | 11 (22.0%) | 92 (47.4%) | 103 (42.2%) |
| III | 12 (24.0%) | 61 (31.4%) | 73 (29.9%) |
| IV | 1 (2.0%) | 16 (8.2%) | 17 (7.0%) |
| Missing | 8 (16.0%) | 0 (0%) | 8 (3.3%) |
| Time |  |  |  |
| Mean (SD) | 26.3 (25.7) | 27.1 (13.9) | 26.9 (16.9) |
| Median [Min, Max] | 20.2 [0, 109] | 26.0 [0.333, 72.0] | 24.0 [0, 109] |
| Event |  |  |  |
| Alive | 20 (40.0%) | 156 (80.4%) | 176 (72.1%) |
| Dead | 30 (60.0%) | 38 (19.6%) | 68 (27.9%) |
