# Supplementary material for: Diagnostic and prognostic values of pyroptosis-related genes for the hepatocellular carcinoma
Source: BMC Bioinformatics. 2022 May 13;23:177. doi: 10.1186/s12859-022-04726-7 (PMC9101834; doi:10.1186/s12859-022-04726-7)
Supplement: Supplementary file 2 — Additional file 2. Table S2. The clinical information of HCC samples in the TCGA cohort and the ICGC cohort. [file 12859_2022_4726_MOESM2_ESM.docx]

Table S2 The clinical information of HCC samples in the TCGA cohort and the ICGC cohort.

| Characteristics | TCGA | |  | | ICGC | | |  | | Total | | | | |
| --- | --- | --- | --- | --- | --- | --- | --- | --- | --- | --- | --- | --- | --- | --- |
|  | Alive | Dead |  | | Alive | | Dead |  | | | Alive | Dead | |  |
|  | (N=234) | (N=77) | | (N=189) | | (N=42) | | | (N=423) | | | | (N=119) | |
| Gender |  |  | |  | |  | | |  | | | |  | |
| Male | 164 (70.1%) | 44 (57.1%) | | 144 (76.2%) | | 26 (61.9%) | | | 308 (72.8%) | | | | 70 (58.8%) | |
| Female | 70 (29.9%) | 33 (42.9%) | | 45 (23.8%) | | 16 (38.1%) | | | 115 (27.2%) | | | | 49 (41.2%) | |
| Age |  |  | |  | |  | | |  | | | |  | |
| Mean (SD) | 59.3 (13.0) | 63.3 (13.1) | | 67.3 (10.3) | | 67.2 (9.55) | | | 62.9 (12.5) | | | | 64.7 (12.1) | |
| Median [Min, Max] | 61.0 [17.0, 85.0] | 65.0 [24.0, 86.0] | | 69.0 [31.0, 89.0] | | 68.5 [37.0, 83.0] | | | 65.0 [17.0, 89.0] | | | | 67.0 [24.0, 86.0] | |
| Missing | 0 (0%) | 1 (1.3%) | | 0 (0%) | | 0 (0%) | | | 0 (0%) | | | | 1 (0.8%) | |
| Age_group |  |  | |  | |  | | |  | | | |  | |
| Younger <65 | 146 (62.4%) | 40 (51.9%) | | 68 (36.0%) | | 14 (33.3%) | | | 214 (50.6%) | | | | 54 (45.4%) | |
| Older >65 | 88 (37.6%) | 36 (46.8%) | | 121 (64.0%) | | 28 (66.7%) | | | 209 (49.4%) | | | | 64 (53.8%) | |
| Missing | 0 (0%) | 1 (1.3%) | | 0 (0%) | | 0 (0%) | | | 0 (0%) | | | | 1 (0.8%) | |
| Family_history |  |  | |  | |  | | |  | | | |  | |
| NO | 139 (59.4%) | 33 (42.9%) | | 0 (0%) | | 0 (0%) | | | 139 (32.9%) | | | | 33 (27.7%) | |
| YES | 61 (26.1%) | 38 (49.4%) | | 0 (0%) | | 0 (0%) | | | 61 (14.4%) | | | | 38 (31.9%) | |
| Missing | 34 (14.5%) | 6 (7.8%) | | 189 (100%) | | 42 (100%) | | | 223 (52.7%) | | | | 48 (40.3%) | |
| Inflammation_grade |  |  | |  | |  | | |  | | | |  | |
| G1 | 30 (12.8%) | 8 (10.4%) | | 0 (0%) | | 0 (0%) | | | 30 (7.1%) | | | | 8 (6.7%) | |
| G2 | 115 (49.1%) | 35 (45.5%) | | 0 (0%) | | 0 (0%) | | | 115 (27.2%) | | | | 35 (29.4%) | |
| G3 | 81 (34.6%) | 28 (36.4%) | | 0 (0%) | | 0 (0%) | | | 81 (19.1%) | | | | 28 (23.5%) | |
| G4 | 7 (3.0%) | 5 (6.5%) | | 0 (0%) | | 0 (0%) | | | 7 (1.7%) | | | | 5 (4.2%) | |
| Missing | 1 (0.4%) | 1 (1.3%) | | 189 (100%) | | 42 (100%) | | | 190 (44.9%) | | | | 43 (36.1%) | |
| Stage |  |  | |  | |  | | |  | | | |  | |
| I | 128 (54.7%) | 38 (49.4%) | | 35 (18.5%) | | 1 (2.4%) | | | 163 (38.5%) | | | | 39 (32.8%) | |
| II | 61 (26.1%) | 16 (20.8%) | | 88 (46.6%) | | 17 (40.5%) | | | 149 (35.2%) | | | | 33 (27.7%) | |
| III | 44 (18.8%) | 20 (26.0%) | | 56 (29.6%) | | 15 (35.7%) | | | 100 (23.6%) | | | | 35 (29.4%) | |
| IV | 1 (0.4%) | 3 (3.9%) | | 10 (5.3%) | | 9 (21.4%) | | | 11 (2.6%) | | | | 12 (10.1%) | |
| Time |  |  | |  | |  | | |  | | | |  | |
| Mean (SD) | 21.6 (23.4) | 24.0 (25.3) | | 29.1 (13.1) | | 17.8 (14.0) | | | 24.9 (19.8) | | | | 21.8 (22.1) | |
| Median [Min, Max] | 12.3 [0.100, 123] | 15.1 [0.300, 109] | | 29.0 [3.00, 72.0] | | 16.0 [0.333, 48.0] | | | 21.0 [0.100, 123] | | | | 15.6 [0.300, 109] | |
| Riskscore |  |  | |  | |  | | |  | | | |  | |
| Mean (SD) | -0.0680 (0.528) | 0.207 (0.623) | | -0.00247 (0.557) | | 0.331 (0.625) | | | -0.0387 (0.542) | | | | 0.251 (0.624) | |
| Median [Min, Max] | -0.136 [-1.34, 1.68] | 0.219 [-0.903, 2.09] | | -0.0199 [-0.997, 2.48] | | 0.247 [-0.983, 1.81] | | | -0.104 [-1.34, 2.48] | | | | 0.219 [-0.983, 2.09] | |
| Risk group |  |  | |  | |  | | |  | | | |  | |
| Low-risk | 185 (79.1%) | 43 (55.8%) | | 166 (87.8%) | | 27 (64.3%) | | | 351 (83.0%) | | | | 70 (58.8%) | |
| High-risk | 49 (20.9%) | 34 (44.2%) | | 23 (12.2%) | | 15 (35.7%) | | | 72 (17.0%) | | | | 49 (41.2%) | |
