# Supplementary material for: Diagnostic and prognostic values of pyroptosis-related genes for the hepatocellular carcinoma
Source: BMC Bioinformatics. 2022 May 13;23:177. doi: 10.1186/s12859-022-04726-7 (PMC9101834; doi:10.1186/s12859-022-04726-7)
Supplement: Supplementary file 3 — Additional file 3. Table S3. Pyroptosis-related genes. [file 12859_2022_4726_MOESM3_ESM.docx]

**Table S3.** Pyroptosis-related genes (PRGs)

| Genes | Full-names | |  |  |  |  |
| --- | --- | --- | --- | --- | --- | --- |
| BAK1 | BCL2 Antagonist/Killer 1 | | |  |  |  |
| BAX | BCL2 Associated X, Apoptosis Regulator | | | | |  |
| CASP1 | Caspase 1 | |  |  |  |  |
| CASP3 | Caspase 3 | |  |  |  |  |
| CASP4 | Caspase 4 | |  |  |  |  |
| CASP5 | Caspase 5 | |  |  |  |  |
| CHMP2A | Charged Multivesicular Body Protein 2A | | | | |  |
| CHMP2B | Charged Multivesicular Body Protein 2B | | | | |  |
| CHMP3 | Charged Multivesicular Body Protein 3 | | | | |  |
| CHMP4A | Charged Multivesicular Body Protein 4A | | | | |  |
| CHMP4B | Charged Multivesicular Body Protein 4B | | | | |  |
| CHMP4C | Charged Multivesicular Body Protein 4C | | | | |  |
| CHMP6 | Charged Multivesicular Body Protein 6 | | | | |  |
| CHMP7 | Charged Multivesicular Body Protein 7 | | | | |  |
| CYCS | Cytochrome C, Somatic | | |  |  |  |
| ELANE | Elastase, Neutrophil Expressed | | | |  |  |
| GSDMD | Gasdermin D | |  |  |  |  |
| GZMB | Granzyme B | |  |  |  |  |
| HMGB1 | High Mobility Group Box 1 | | |  |  |  |
| IL18 | Interleukin 18 | |  |  |  |  |
| IL1A | Interleukin 1 Alpha | | |  |  |  |
| IL1B | Interleukin 1 Beta | | |  |  |  |
| IRF1 | Interferon Regulatory Factor 1 | | | |  |  |
| IRF2 | Interferon Regulatory Factor 2 | | | |  |  |
| TP53 | Tumor Protein P53 | |  |  |  |  |
| TP63 | Tumor Protein P63 | |  |  |  |  |
| NLRP3 | NLR Family Pyrin Domain Containing 3 | | | | |  |
| GSDMB | Gasdermin B | |  |  |  |  |
| AIM2 | Absent In Melanoma 2 | | |  |  |  |
| CASP6 | Caspase 6 | |  |  |  |  |
| CASP8 | Caspase 8 | |  |  |  |  |
| CASP9 | Caspase 9 | |  |  |  |  |
| GPX4 | Glutathione Peroxidase 4 | | |  |  |  |
| GSDMA | Gasdermin A | |  |  |  |  |
| GSDMC | Gasdermin C | |  |  |  |  |
| GSDME | Gasdermin E | |  |  |  |  |
| IL6 | Interleukin 6 | |  |  |  |  |
| NLRC4 | NLR Family CARD Domain Containing 4 | | | |  |  |
| NLRP1 | NLR Family Pyrin Domain Containing 1 | | | | |  |
| NLRP2 | NLR Family Pyrin Domain Containing 2 | | | | |  |
| NLRP6 | NLR Family Pyrin Domain Containing 6 | | | | |  |
| NLRP7 | NLR Family Pyrin Domain Containing 7 | | | | |  |
| NOD1 | Nucleotide Binding Oligomerization Domain Containing 1 | | | | | |
| NOD2 | Nucleotide Binding Oligomerization Domain Containing 2 | | | | | |
| PLCG1 | Phospholipase C Gamma 1 | | |  |  |  |
| PRKACA | Protein Kinase CAMP-Activated Catalytic Subunit Alpha | | | | | |
| PYCARD | PYD And CARD Domain Containing | | | |  |  |
| SCAF11 | SR-Related CTD Associated Factor 11 | | | |  |  |
| TIRAP | TIR Domain Containing Adaptor Protein | | | | |  |
| TNF | Tumor Necrosis Factor | | |  |  |  |
| PJVK | Pejvakin |  |  |  |  |  |
