# Supplementary material for: Diagnostic and prognostic values of pyroptosis-related genes for the hepatocellular carcinoma
Source: BMC Bioinformatics. 2022 May 13;23:177. doi: 10.1186/s12859-022-04726-7 (PMC9101834; doi:10.1186/s12859-022-04726-7)
Supplement: Supplementary file 6 — Additional file 6. Table S6. The clinical information of the IMvirgor210 cohort. [file 12859_2022_4726_MOESM6_ESM.docx]

Table S6 The clinical information of the IMvirgor210 cohort.

| Characteristics | Alive | Dead | Total |
| --- | --- | --- | --- |
|  | (N=116) | (N=232) | (N=348) |
| Sex |  |  |  |
| Male | 95 (81.9%) | 177 (76.3%) | 272 (78.2%) |
| Female | 21 (18.1%) | 55 (23.7%) | 76 (21.8%) |
| Response |  |  |  |
| CR | 24 (20.7%) | 1 (0.4%) | 25 (7.2%) |
| PR | 39 (33.6%) | 4 (1.7%) | 43 (12.4%) |
| SD | 23 (19.8%) | 40 (17.2%) | 63 (18.1%) |
| PD | 23 (19.8%) | 144 (62.1%) | 167 (48.0%) |
| Missing | 7 (6.0%) | 43 (18.5%) | 50 (14.4%) |
| Time |  |  |  |
| Mean (SD) | 18.0 (5.82) | 6.35 (5.05) | 10.2 (7.66) |
| Median [Min, Max] | 20.1 [0.197, 24.5] | 5.03 [0.230, 21.2] | 8.05 [0.197, 24.5] |
| Riskscore |  |  |  |
| Mean (SD) | -1.45 (0.810) | -1.25 (0.753) | -1.31 (0.777) |
| Median [Min, Max] | -1.62 [-3.24, 1.04] | -1.35 [-2.76, 1.51] | -1.47 [-3.24, 1.51] |
| Risk group |  |  |  |
| Low-risk | 65 (56.0%) | 94 (40.5%) | 159 (45.7%) |
| High-risk | 51 (44.0%) | 138 (59.5%) | 189 (54.3%) |

Note: CR: complete response, PR: partial response, SD: stable disease, PD: progressive disease.
